# From obesity to cancer: Gut microbiome mechanisms, biomarkers, and U.S. public health strategies

**DOI:** 10.18632/oncoscience.634

**Published:** 2025-11-07

**Authors:** Hashim Muhammad Moseeb, Mohsin Muhammad Aizaz, Khan Aiza, Thakur Hammed Hafsa, Muzaffar Sania, Zahoor Kamran, Zahra Tu Shamama, Ashraf Muhammad Usama, Qureshi Pir Maroof, Fatima Feroze, Rahu Ahmed, Naeem Ammara, Gandhi Mahima

**Affiliations:** ^1^University of Missouri-Columbia, Columbia, MO 65201, USA; ^2^School of Medicine, University of Buckingham, Buckingham, UK; ^3^Research Associate, Alpha Clinical Developments Ltd., UK; ^4^Department of Pathology, Dow University of Health Sciences, Karachi, Sindh 74200, Pakistan; ^5^Lahore General Hospital, Lahore 54000, Pakistan; ^6^Excellent Medical Associates, Chicago, IL 60462, USA; ^7^Department of Pathology, Liaquat University hospital, Hyderabad 71000, Pakistan; ^8^Department of Pathology, Primary Health Care Corporation, Qatar; ^9^Department of Pathology, University of Toledo Medical Center, OH 43606, USA; ^10^Department of Medicine, Pakistan Kidney Patient’s Association, Islamabad, Pakistan; ^11^Department of Pathology, Dr Ziauddin Hospital Karachi, Karachi 74700, Pakistan

**Keywords:** gut microbiome, obesity, metabolic syndrome, colorectal cancer, dysbiosis

## Abstract

Background: Obesity, metabolic syndrome, and colorectal cancer (CRC) remain major public health challenges in the United States, collectively driving substantial morbidity, mortality, and economic burden. Beyond diet and genetics, the gut microbiome has emerged as a pivotal determinant of host metabolism, immunity, and carcinogenesis, influenced by both environmental and behavioral factors.

Objective: This review synthesizes current evidence linking gut microbial dysbiosis to obesity, metabolic syndrome, and CRC, emphasizing mechanistic pathways, environmental modifiers, and translational opportunities relevant to U.S. public health and precision medicine.

Methods: Comprehensive searches of PubMed and Scopus (2000–2025) identified large epidemiologic studies, mechanistic experiments, and clinical trials, prioritizing research from U.S. populations and nationally representative databases including NHANES, SEER, and the Nurses’ Health Study.

Results: Microbial alterations such as enrichment of Fusobacterium nucleatum, enterotoxigenic Bacteroides fragilis, and colibactin-producing Escherichia coli contribute to CRC initiation and progression. In obesity and metabolic syndrome, shifts in Firmicutes-to-Bacteroidetes ratios, altered short-chain fatty acid metabolism, and endotoxin-mediated inflammation disrupt metabolic homeostasis. Environmental and lifestyle exposures, including air pollutants, smoking, and Westernized diets, modulate microbial ecology across the aerodigestive tract, affecting disease susceptibility. The emerging discipline of Molecular Pathological Epidemiology (MPE) integrates lifestyle, microbiome, and biomarker data to elucidate exposure-outcome relationships, enabling personalized prevention and therapeutic strategies.

Conclusions: The gut microbiome functions as both a biomarker and therapeutic target across metabolic and neoplastic diseases. Integrating microbiome science with environmental epidemiology and MPE frameworks offers transformative potential for precision prevention and equitable public health strategies in the U.S.

## INTRODUCTION

Obesity, metabolic syndrome, and colorectal cancer (CRC) remain among the most pressing public health challenges in the United States. Obesity affects more than 40% of U.S. adults, contributing substantially to the burden of type 2 diabetes, cardiovascular disease, and cancer risk [1, 2]. Similarly, CRC is the third most commonly diagnosed cancer and the second leading cause of cancer death in the U.S., with incidence trends strongly linked to dietary and lifestyle factors [3, 4]. These conditions represent major sources of health care expenditure and mortality, underscoring the urgent need for novel approaches to prevention and management.

In recent years, the gut microbiome has emerged as a pivotal factor in host metabolism, immunity, and carcinogenesis. Alterations in gut microbial composition—often termed “dysbiosis”—have been consistently associated with obesity, insulin resistance, and chronic low-grade inflammation [5–7]. Beyond metabolic disorders, mounting evidence suggests a role for the microbiome in CRC initiation and progression, particularly through the activity of species such as *Fusobacterium nucleatum*, enterotoxigenic *Bacteroides fragilis*, and colibactin-producing *Escherichia coli* [8, 9]. Mechanistic pathways include modulation of host immune responses, production of microbial metabolites such as short-chain fatty acids and secondary bile acids, and direct genotoxic effects [10].

Importantly, large U.S. cohort studies have begun to integrate microbiome profiling with longitudinal health outcomes. Analyses from the Nurses’ Health Study and the Health Professionals Follow-up Study demonstrate that specific microbial signatures correlate with CRC risk and obesity phenotypes [11]. Parallel findings from NHANES-linked microbiome investigations reinforce the national relevance of gut microbial shifts in shaping metabolic health [12]. These discoveries have spurred translational efforts toward microbiome-based diagnostics, dietary interventions, and therapeutic approaches, including fecal microbiota transplantation and engineered probiotics.

Given the rising prevalence of obesity and CRC in the U.S., coupled with advances in microbiome science, a comprehensive synthesis of current knowledge is warranted. This review aims to (1) summarize the role of the gut microbiome in obesity, metabolic syndrome, and CRC, (2) highlight mechanistic pathways linking microbes to host metabolism and carcinogenesis, and (3) evaluate translational and public health implications in the U.S. context.

## THE GUT MICROBIOME IN OBESITY AND METABOLIC SYNDROME

Obesity and metabolic syndrome represent intertwined conditions that impose a significant burden on the U.S. health care system. More than 40% of American adults meet criteria for obesity, and approximately one in three meet criteria for metabolic syndrome, reflecting a convergence of dyslipidemia, insulin resistance, central adiposity, and hypertension [13]. While excess caloric intake and sedentary lifestyle remain central drivers, the gut microbiome has emerged as a crucial mediator of host energy balance, nutrient metabolism, and inflammatory tone.

### Altered microbial composition

Studies consistently demonstrate altered microbial composition in obese individuals, with an increased Firmicutes-to-Bacteroidetes ratio often observed [5, 14]. This microbial shift appears to enhance the extraction of energy from otherwise indigestible polysaccharides, thereby contributing to increased adiposity. Landmark metagenomic work by Turnbaugh et al. [5] showed that obese individuals harbor gut microbiomes with increased metabolic capacity for harvesting energy, a finding later confirmed in U.S. twin studies [6]. However, not all studies reproduce the Firmicutes–Bacteroidetes paradigm, suggesting that functional pathways, rather than taxonomy alone, may be more critical to host metabolic outcomes. These inconsistencies highlight the heterogeneity of human microbiomes across different ethnic, dietary, and geographic backgrounds. Future studies are needed to reconcile these differences and identify universal versus population-specific microbial patterns.

### Short-chain fatty acids and host metabolism

Short-chain fatty acids (SCFAs), primarily acetate, propionate, and butyrate, represent key microbial metabolites linking the gut microbiome to metabolic health. SCFAs regulate appetite, improve gut barrier function, and modulate insulin sensitivity via G-protein–coupled receptors [15]. In obesity, SCFA profiles may be altered, with increased acetate linked to lipogenesis and reduced butyrate associated with impaired gut barrier integrity [16]. In U.S. human cohorts, SCFA concentrations have been correlated with insulin resistance and visceral adiposity [17], underscoring their role as mechanistic mediators. Beyond metabolic signaling, SCFAs also influence central nervous system function through the gut–brain axis, contributing to appetite control and mood regulation. Disruption of this pathway may exacerbate the behavioral and psychological components of obesity [18].

### Microbiome-driven inflammation and insulin resistance

Low-grade systemic inflammation is a hallmark of metabolic syndrome. Dysbiosis can promote endotoxemia through increased abundance of Gram-negative bacteria and elevated circulating lipopolysaccharides (LPS) [19]. This process activates Toll-like receptor 4 (TLR4) signaling, contributing to insulin resistance in adipose and hepatic tissues. Murine models colonized with “obese microbiota” develop increased adiposity and inflammation compared with lean microbiota–transplanted controls [20]. In humans, endotoxin-associated inflammation has been shown to mediate the link between dysbiosis and insulin resistance [21]. Such inflammation also promotes endothelial dysfunction, linking microbiome changes to cardiovascular risk in metabolic syndrome [22]. These findings reinforce the concept that the gut microbiome acts as both a metabolic and vascular regulator.

### Evidence from U.S. cohort studies

Several U.S.-based studies have highlighted the population-level relevance of microbiome–metabolic interactions. In the Hispanic Community Health Study/Study of Latinos, specific bacterial taxa were linked to obesity and metabolic traits across diverse populations [23]. Similarly, data from the Multi-Ethnic Study of Atherosclerosis (MESA) revealed associations between microbial diversity and insulin sensitivity [24]. These findings suggest that microbiome alterations may help explain ethnic disparities in obesity prevalence and outcomes in the United States. These cohort-based insights are particularly valuable for shaping targeted public health interventions in diverse U.S. populations. Integrating microbiome data into longitudinal epidemiologic studies will clarify causal pathways and inform prevention strategies.

### GUT MICROBIOTA AND COLORECTAL CANCER

Colorectal cancer (CRC) remains the second leading cause of cancer-related death in the United States, with an estimated 151,030 new cases and 52,580 deaths projected in 2022 [3, 25]. While genetic predisposition and lifestyle factors such as diet and obesity play key roles, increasing evidence suggests that the gut microbiome contributes to colorectal carcinogenesis through complex host–microbe interactions [26]. Dysbiosis not only promotes chronic mucosal inflammation but also drives tumor initiation and progression through microbial metabolites, direct genotoxins, and modulation of host immune responses.

### Microbial taxa implicated in CRC

Several bacterial species have been strongly linked to CRC pathogenesis as shown in Table 1. *Fusobacterium nucleatum* has been shown to promote tumor growth by stimulating inflammatory responses and suppressing anti-tumor immunity [27]. Enterotoxigenic *Bacteroides fragilis* produces a metalloprotease toxin that activates Wnt and NF-κB signaling, fostering epithelial proliferation [28]. Colibactin-producing *Escherichia coli* can directly induce DNA damage and chromosomal instability [29]. Metagenomic analyses consistently identify enrichment of these taxa in tumor-associated microbiota compared with normal controls, suggesting potential diagnostic applications. The enrichment of these species is not random but reflects selective pressures created by the tumor microenvironment. Understanding these microbial–tumor interactions could lead to development of more refined biomarkers for CRC detection [30].

**Table 1:** Key gut microbial taxa and their roles in obesity and colorectal cancer

Microbial taxa	Role in obesity/metabolic syndrome	Role in colorectal cancer (CRC)	Mechanisms of action
**Bacteroidetes (Decreased)**	Decreased abundance in obesity	Protective taxa reduced in CRC	Lower SCFA production, altered gut ecology
**Fusobacterium nucleatum**	Not strongly linked	Enriched in CRC; promotes tumor progression	Immune evasion, inflammation
**Bacteroides fragilis (ETBF)**	Possible dysbiosis contributor	Enterotoxigenic strains associated with CRC	Toxin activates NF-κB and Wnt signaling
**Escherichia coli (pks+)**	Dysbiosis-related endotoxemia	Colibactin-producing strains cause DNA damage	Genotoxicity, genomic instability
**Sulfate-reducing bacteria**	Not directly linked	Produce hydrogen sulfide, a genotoxic metabolite	DNA damage, mucosal injury

Abbreviations: SCFA: Short-Chain Fatty Acids; ETBF: Enterotoxigenic *Bacteroides fragilis*; NF-κB: Nuclear Factor Kappa-Light-Chain-Enhancer of Activated B Cells; Wnt: Wingless/Integrated signaling pathway.

### Microbial metabolites and carcinogenesis

Beyond taxonomy, microbial metabolites play a central role in carcinogenesis. Secondary bile acids such as deoxycholic acid, elevated in high-fat Western diets, can induce oxidative stress and DNA damage [31]. Hydrogen sulfide, produced by sulfate-reducing bacteria, exerts genotoxic effects on colonocytes [32]. Conversely, short-chain fatty acids like butyrate demonstrate protective properties by promoting epithelial differentiation and inducing apoptosis of malignant cells [33]. Thus, the balance of microbial metabolites can determine pro- versus anti-carcinogenic environments in the colon. These findings highlight how dietary exposures shape cancer risk indirectly through microbial metabolism. This also underscores why lifestyle interventions may be as important as pharmacologic therapies in CRC prevention [34].

### Mechanistic insights

Mechanistic studies have elucidated several host pathways altered by gut microbiota in CRC. Microbial products activate Toll-like receptors (TLRs) and downstream NF-κB signaling, promoting inflammation-driven tumorigenesis [35]. Dysbiosis can alter epigenetic programming via histone acetylation and DNA methylation [36]. Mouse models colonized with CRC-associated bacteria develop accelerated tumor growth, supporting a causal relationship [37]. These mechanistic insights also highlight potential therapeutic targets such as TLR inhibition and epigenetic reprogramming. By linking microbial presence with functional pathways, they provide a bridge between observational findings and translational interventions [38].

### Evidence from U.S. cohort studies

Large prospective U.S. cohorts have begun to integrate microbiome data with cancer risk. In the Nurses’ Health Study and Health Professionals Follow-up Study, dietary patterns promoting dysbiosis such as high consumption of processed meat and refined grains were associated with increased CRC risk, particularly in tumors enriched with *Fusobacterium nucleatum* [39]. Similarly, tissue-based analyses revealed that microbial signatures may distinguish CRC subtypes, underscoring potential for microbiome-informed risk stratification [40]. These findings highlight the relevance of microbiome alterations not only for understanding pathogenesis but also for guiding precision prevention strategies.

## MECHANISTIC INSIGHTS

**Figure 1:**
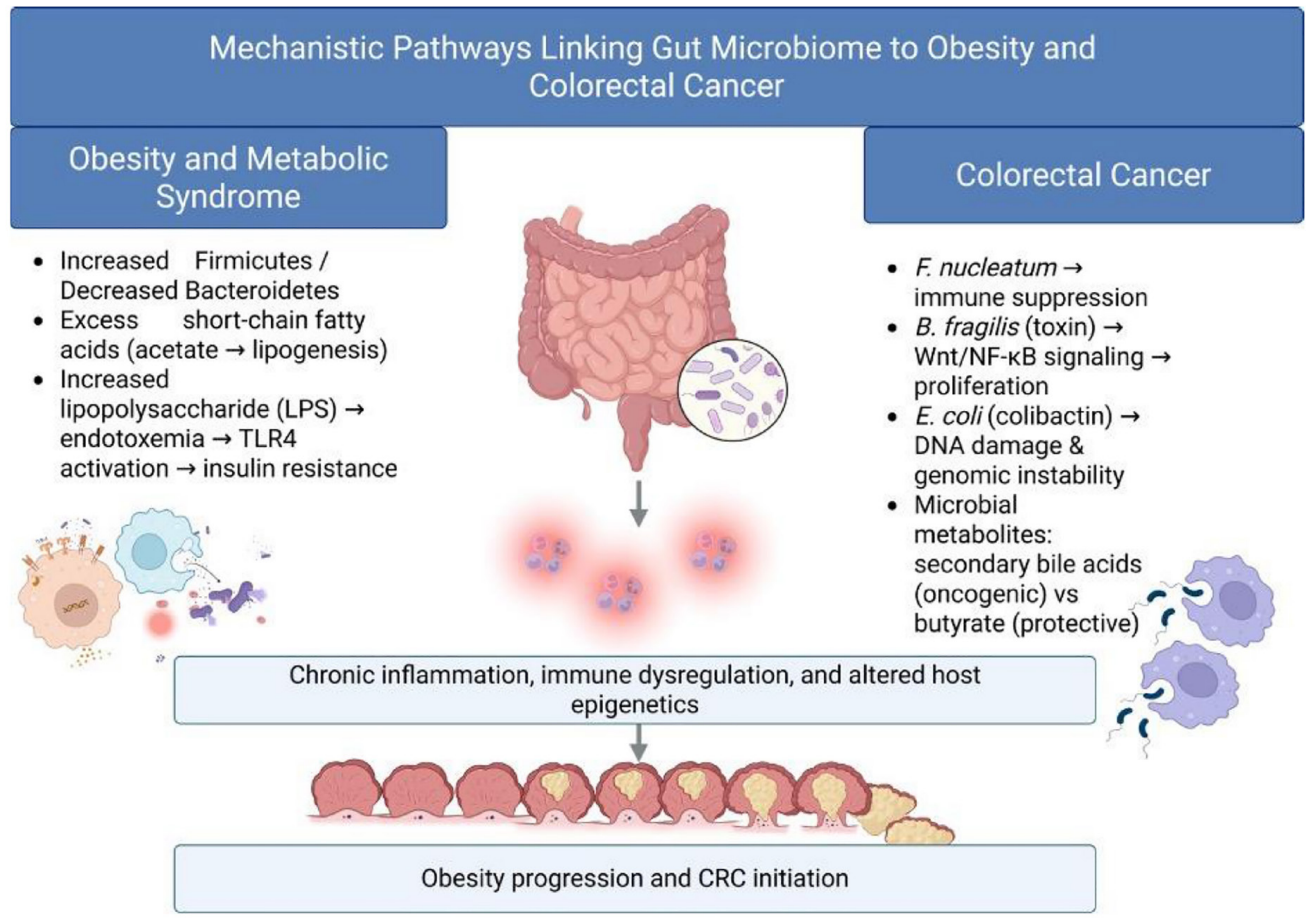
Mechanistic pathways linking gut microbiome to obesity and CRC. Gut dysbiosis promotes obesity and CRC through microbial composition shifts, metabolite imbalance, endotoxemia, and immune modulation. Certain taxa exert pro-tumorigenic effects, while protective metabolites (e.g., butyrate) are diminished. Abbreviations: LPS: Lipopolysaccharide; TLR4: Toll-Like Receptor 4; NF-KB: Nuclear Factor KappaLight-Chain-Enhancer of Activated B Cells; Wnt: Wingless/Integrated signaling pathway; SCFAs: Short-Chain Fatty Acids (acetate, propionate, butyrate).

The gut microbiome influences colorectal carcinogenesis and metabolic disease not only through microbial composition but also via multiple mechanistic pathways that shape host immunity, metabolism, and gene regulation as shown in Figure 1. Understanding these processes provides critical insight into how dysbiosis may translate into disease phenotypes observed in the U.S. population.

### Host–microbe immune interactions

The intestinal epithelium serves as both a physical and immunological barrier. Microbial products such as lipopolysaccharide (LPS), flagellin, and peptidoglycan interact with Toll-like receptors (TLRs) and NOD-like receptors, activating downstream pathways including NF-κB and MAPK [41]. Chronic activation of these pathways results in increased secretion of proinflammatory cytokines such as IL-6, TNF-α, and IL-17, which sustain a pro-tumorigenic microenvironment [42]. In mouse models, IL-23/IL-17 signaling driven by microbial stimulation has been shown to accelerate colorectal tumor growth [37]. Importantly, immune responses triggered by dysbiosis may not remain localized to the gut but can spill over systemically, influencing obesity-related metabolic inflammation. These findings highlight the immune system as a shared mediator linking the microbiome to multiple chronic diseases [43].

### Microbial metabolites and epigenetic regulation

Microbial metabolites exert profound epigenetic effects on host cells. Short-chain fatty acids (SCFAs), particularly butyrate, act as histone deacetylase (HDAC) inhibitors, thereby modulating gene transcription [44, 45]. Butyrate can promote apoptosis in cancer cells while supporting epithelial barrier integrity in healthy tissue. Conversely, genotoxic metabolites such as colibactin and secondary bile acids induce DNA strand breaks and alter chromatin stability, fostering mutagenesis [46]. These mechanisms illustrate how metabolites shape the balance between protective and carcinogenic outcomes. The dual nature of these metabolites also suggests therapeutic potential in selectively amplifying protective microbial pathways. Harnessing this knowledge could pave the way for diet-based or pharmacologic interventions aimed at epigenetic reprogramming [47].

### DNA damage and genomic instability

Direct microbial genotoxins have been implicated in CRC pathogenesis. Colibactin-producing *Escherichia coli* induces interstrand cross-links and double-strand DNA breaks, resulting in chromosomal instability [48]. This activity can cooperate with inflammation-driven oxidative stress to accelerate tumor initiation. In murine models, colonization with colibactin-positive *E. coli* significantly increases tumor burden compared to colonization with non-toxigenic strains [48]. These findings suggest that certain microbes may function as true carcinogens rather than just promoters of inflammation. Targeting such high-risk bacterial strains could form the basis of precision prevention strategies in CRC [49].

### Metabolic and endocrine modulation

The gut microbiome modulates host metabolic signaling through bile acid receptors (FXR, TGR5), G-protein–coupled receptors (GPR41, GPR43), and aryl hydrocarbon receptors [50]. Dysregulated signaling contributes to altered lipid metabolism, insulin resistance, and pro-carcinogenic bile acid profiles. Importantly, Western dietary patterns prevalent in the U.S. enhance bile acid–producing microbial populations, linking national dietary habits to increased CRC risk [51]. This endocrine crosstalk illustrates how the microbiome extends its influence beyond the gut, affecting systemic hormonal and metabolic regulation. Such mechanisms provide a rationale for including microbiome endpoints in national dietary and lifestyle intervention trials [52].

### Integration of multi-omics approaches

Recent U.S. studies integrating metagenomics, metabolomics, and transcriptomics provide comprehensive views of microbiome–host interactions. These analyses highlight that functional microbial pathways, rather than taxonomic composition alone, may be the critical determinants of disease risk [48]. Such multi-omics strategies are essential for developing microbiome-informed diagnostics and personalized prevention strategies. Integrating multi-omics with electronic health records and large-scale biobanks will accelerate translation into precision public health. The ability to predict disease risk through integrated datasets could transform screening and prevention paradigms.

### Environmental and lifestyle modifiers of microbial pathophysiology

Beyond host genetics and microbial composition, environmental and lifestyle factors profoundly influence the microbiome and associated disease mechanisms. Air pollution, smoking, and occupational exposures can disrupt microbial communities throughout the aerodigestive tract, promoting inflammation and carcinogenesis [53, 54]. Dietary habits, physical activity, alcohol consumption, and sleep patterns likewise shape microbial metabolism, immune tone, and epithelial barrier integrity. These exposures act synergistically with microbial metabolites to alter cellular signaling, oxidative stress, and DNA repair [55]. Differences in environmental and behavioral exposures among individuals may partially explain variations in disease risk, outcomes, and response to microbiome-targeted therapies [56]. Understanding these modifiers is essential for designing personalized prevention strategies that integrate microbiome science with environmental health.

### Molecular pathological epidemiology (MPE): An integrative framework

Recent advances highlight the emerging field of Molecular Pathological Epidemiology (MPE), which integrates molecular pathology with epidemiologic and bioinformatic approaches to understand how lifestyle, environmental, and genetic factors interact to drive disease heterogeneity [57]. MPE studies link specific exposures such as diet, obesity, smoking, and microbiome composition to molecular tumor subtypes and treatment responses [58]. This integrative approach provides a powerful framework to study the biological consequences of exposures within distinct molecular disease contexts.

MPE has been applied in gastrointestinal and colorectal cancer research to evaluate how microbial signatures, immune markers, and mutational profiles jointly influence outcomes and therapy response [59].Incorporating MPE concepts into microbiome research allows investigators to examine how microbial dysbiosis mediates environmental risk factors at a molecular level bridging epidemiology, molecular pathology, and clinical outcomes. Such frameworks will be essential for translating microbiome science into precision prevention and personalized medicine strategies in the U.S. public health landscape.

## MICROBIOME-BASED DIAGNOSTICS AND THERAPEUTICS

As evidence linking the gut microbiome to obesity, metabolic syndrome, and colorectal cancer (CRC) continues to expand, efforts have accelerated toward clinical translation. Advances in sequencing technologies, computational biology, and synthetic microbiology are enabling the development of microbiome-informed diagnostics and therapeutic strategies. Several approaches are now moving from research into clinical practice in the United States, reflecting the field’s national and global significance.

### Microbiome as a diagnostic biomarker

Stool-based microbiome profiling has emerged as a promising noninvasive diagnostic tool for CRC detection. Case–control studies have demonstrated that microbial signatures, particularly enrichment of *Fusobacterium nucleatum* and *Bacteroides fragilis*, can distinguish CRC patients from healthy controls with diagnostic accuracy comparable to fecal immunochemical testing (FIT) [60]. Integration of microbial markers with FIT has been shown to improve sensitivity for early-stage CRC [61]. Metabolomic profiling of short-chain fatty acids (SCFAs), bile acids, and other microbial metabolites further refines risk stratification [62]. While not yet widely adopted in U.S. clinical practice, ongoing studies in large prospective cohorts suggest a future role for microbiome-based screening adjuncts. Incorporating microbial biomarkers into standard CRC screening could reduce false negatives and personalize colonoscopy recommendations. As sequencing costs decline, population-level implementation of such diagnostics may become feasible [63].

### Probiotics, prebiotics, and dietary interventions

Targeted modulation of the gut microbiome through probiotics and prebiotics is a widely explored therapeutic avenue. Clinical trials demonstrate that specific probiotic strains, including *Bifidobacterium* and *Lactobacillus*, may improve insulin sensitivity and reduce markers of inflammation in obesity and metabolic syndrome [64]. Prebiotics such as inulin and resistant starch selectively stimulate beneficial taxa that produce butyrate, a metabolite with anti-carcinogenic properties [65]. Importantly, dietary interventions such as increased fiber and reduced red meat intake have been shown in both U.S. and international cohorts to shift microbial composition toward protective profiles [66, 67]. Beyond metabolic health, such dietary strategies may also influence mood and cognitive function through the gut–brain axis. These multidimensional benefits make dietary modulation a cost-effective and scalable public health strategy [68].

### Fecal microbiota transplantation (FMT)

Fecal microbiota transplantation (FMT) represents the most direct approach to microbiome restoration. Although currently FDA-approved only for recurrent *Clostridioides difficile* infection, exploratory trials are assessing FMT in obesity, insulin resistance, and CRC prevention [69]. Early results suggest transient improvements in insulin sensitivity, although sustained benefits require optimized donor selection and delivery methods [70]. Safety, donor screening, and standardization remain significant regulatory challenges, but the approval of microbiota-based live biotherapeutic products marks a critical step forward. Long-term follow-up of FMT recipients is essential to evaluate durability of clinical effects and potential adverse consequences. Expanding donor diversity may also enhance therapeutic outcomes across different U.S. populations [71].

### FDA-approved microbiota therapeutics

In 2022, the FDA approved the first microbiota-based therapeutic, RBX2660 (Rebyota^®^), for prevention of recurrent *C. difficile* infection [72]. This milestone signals a regulatory pathway for future microbiota-targeted products. Other candidates, including SER-109, an oral microbiota capsule, have demonstrated efficacy in late-phase trials [73]. Beyond infectious diseases, engineered probiotics designed to deliver therapeutic molecules (e.g., immunomodulators, anti-inflammatory peptides) are under investigation in oncology and metabolic disorders [74]. These innovations highlight the rapid transition of microbiome science from bench to bedside. The establishment of this regulatory precedent could accelerate FDA review of future microbiota therapies. This shift reflects a broader recognition that microbial-based interventions are integral to next-generation precision medicine.

### Bacteriophage therapy and precision microbiome engineering

Next-generation strategies aim to selectively deplete pathogenic bacteria while preserving commensals. Bacteriophage therapy targeting CRC-associated taxa such as *Fusobacterium nucleatum* has shown efficacy in preclinical models [75]. CRISPR-based microbial engineering and synthetic biology approaches are also being developed to reprogram gut microbiota for therapeutic benefit. While still experimental, these technologies could provide precision tools for microbiome modulation in high-risk populations. Such approaches offer the potential to overcome the limitations of broad-spectrum antibiotics, which often disrupt protective commensals. If proven safe, phage- and CRISPR-based therapies could transform the treatment of microbiome-associated diseases [76]. Table 2 below shows some of the translational applications of gut microbiome science in US public health.

**Table 2:** Translational applications of gut microbiome science in U.S. public health

Application domain	Examples	Relevance to U.S. public health
Diagnostics	Microbial signatures (e.g., *Fusobacterium* in stool), SCFA/bile acid metabolomics, FIT + microbiome panels	Potential adjunct to CRC screening; improved early detection
Therapeutics	Probiotics, Prebiotics, FMT, FDA-approved microbiota therapeutics (Rebyota^®^, SER-109), engineered probiotics/phages	New treatment strategies for obesity, metabolic syndrome, and CRC
Policy/Public Health	CDC obesity prevention programs, NCI cancer prevention initiatives, U.S. dietary guidelines, equity-focused microbiome studies	Integration of microbiome science into national health strategy

Abbreviations: SCFA: Short-Chain Fatty Acids; FIT: Fecal Immunochemical Test; FMT: Fecal Microbiota Transplantation; FDA: U.S. Food and Drug Administration; CDC: U.S. Centers for Disease Control and Prevention; NCI: U.S. National Cancer Institute.

## PUBLIC HEALTH AND POLICY IMPLICATIONS (U.S. FOCUS)

**Figure 2:**
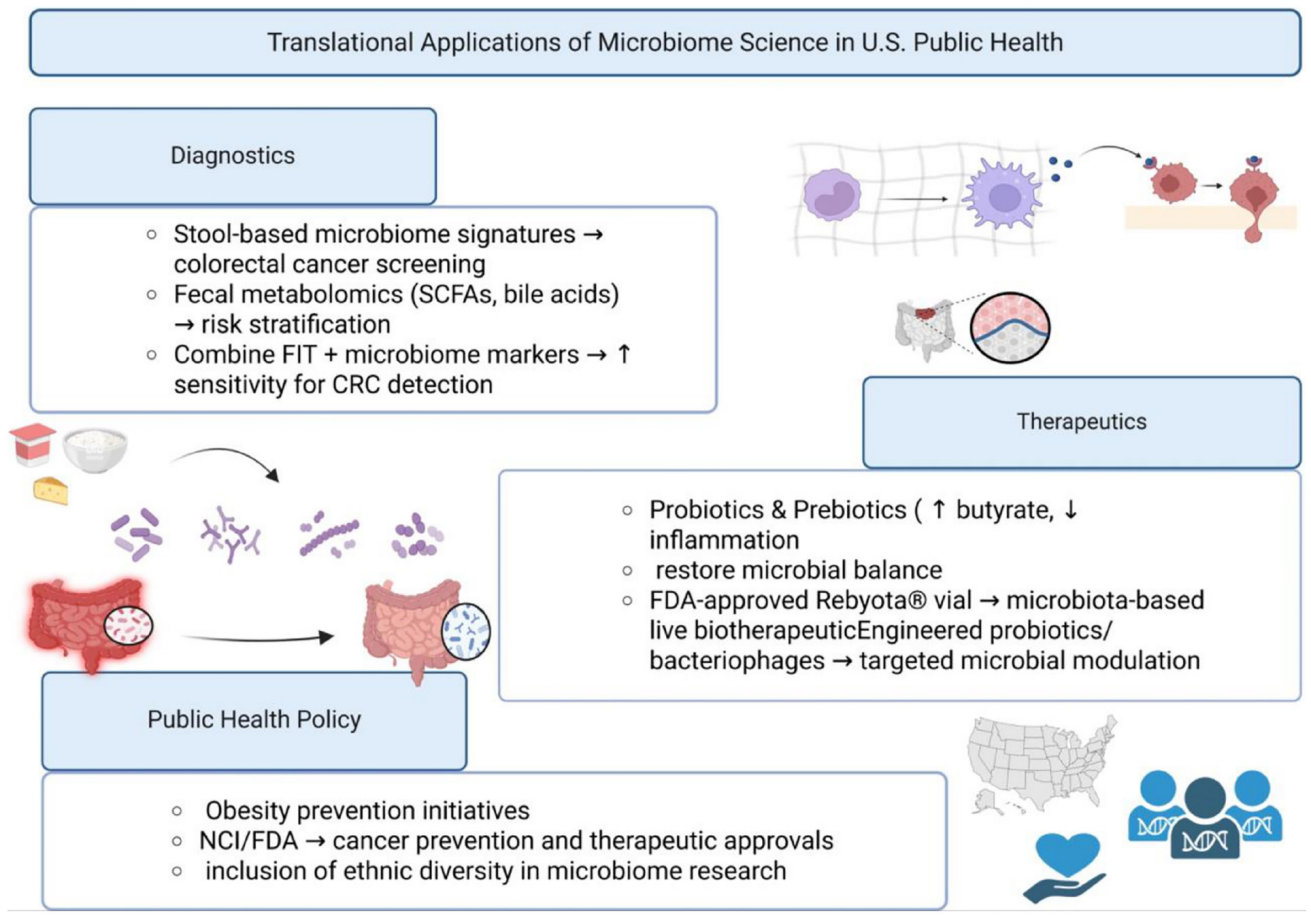
Translational applications of microbiome science in U.S. public health. Emerging applications of microbiome science span from diagnostics (microbial and metabolomic biomarkers) to therapeutics (diet, probiotics, FMT, FDA-approved live biotherapeutics) and integration into U.S. public health policy. Abbreviations: SCFAs: Short-Chain Fatty Acids; FIT: Fecal Immunochemical Test; FMT: Fecal Microbiota Transplantation; FDA: U.S. Food and Drug Administration; CDC: U.S. Centers for Disease Control and Prevention; NCI: U.S. National Cancer Institute.

The rising burden of obesity, metabolic syndrome, and colorectal cancer (CRC) underscores the importance of incorporating microbiome science into U.S. public health strategies. As microbiome-based diagnostics and therapeutics advance, their integration with national programs can enhance disease prevention, reduce disparities, and improve outcomes as shown in Figure 2.

### Epidemiologic significance for the U.S.

Obesity and CRC together represent major drivers of morbidity and mortality. NHANES-based studies show that more than 40% of American adults are obese, with prevalence continuing to rise across racial and ethnic groups [77]. SEER data confirm that CRC remains the second-leading cause of cancer death, with particularly concerning increases among younger adults [78]. These statistics highlight the urgency of innovative preventive strategies, including microbiome-targeted interventions. Without effective interventions, these trends are projected to worsen, adding strain to U.S. healthcare systems. Integrating microbiome-informed strategies into routine practice could help slow or reverse these trajectories [79].

### Dietary policy and microbiome health

Dietary factors represent a key interface between the microbiome and public health. The Western diet, characterized by high fat and low fiber, is associated with dysbiosis, production of carcinogenic metabolites, and obesity-related inflammation [51]. In contrast, fiber-rich diets improve microbial diversity and short-chain fatty acid production, which are protective against CRC and metabolic disease [80]. U.S. dietary guidelines increasingly emphasize whole grains, legumes, fruits, and vegetables — recommendations consistent with microbiome research and relevant to population-level prevention [81]. These guidelines could be further strengthened by explicitly including microbiome outcomes as health benchmarks. Doing so would provide measurable targets to evaluate the effectiveness of dietary policy in real-world populations.

### FDA and regulatory landscape

The FDA approval of Rebyota^®^ and ongoing evaluation of oral microbiota therapeutics demonstrate a clear regulatory pathway for microbiome-based interventions [82]. As these products expand beyond *Clostridioides difficile* infection toward metabolic and oncologic applications, regulatory oversight will be critical to ensure safety, efficacy, and equitable access. The emergence of microbiome-based diagnostics, including stool microbial and metabolomic biomarkers, may also require standardized validation frameworks to support clinical adoption. Establishing harmonized regulatory standards will help accelerate innovation while maintaining public trust. Greater collaboration between the FDA, NIH, and industry stakeholders could streamline the transition of these products into clinical use.

### Addressing health disparities

Disparities in obesity and CRC incidence across racial and socioeconomic groups highlight the need for inclusive microbiome research. African American and Hispanic populations face disproportionate burdens of obesity and CRC [83]. Early evidence suggests that differences in microbiome composition may partially contribute to these disparities [84]. Ensuring diverse representation in U.S. microbiome studies is essential for developing equitable diagnostics and therapeutics that benefit all populations. Community engagement and culturally tailored interventions will be critical to ensure that emerging microbiome strategies are accessible and effective for minority populations. Addressing disparities now may prevent widening health gaps in the era of precision medicine.

### Integration with national programs

Integration of microbiome-informed strategies into existing U.S. programs offers opportunities for high impact. For example CDC’s obesity prevention initiatives could incorporate microbiome research into dietary policy. NCI’s cancer prevention programs may leverage microbial biomarkers for early CRC detection. NIH’s All of Us Research Program offers a platform for integrating microbiome, genomic, and lifestyle data at scale. Such initiatives demonstrate how microbiome research aligns directly with U.S. national health priorities. Embedding microbiome endpoints into these programs would create a robust infrastructure for long-term population surveillance. This integration could also accelerate discovery of actionable biomarkers with direct relevance to public health practice.

## FUTURE DIRECTIONS AND RESEARCH GAPS

Despite major advances in understanding the gut microbiome’s role in obesity, metabolic syndrome, and colorectal cancer (CRC), significant challenges remain before these findings can be fully translated into clinical and public health practice. Addressing these gaps will be critical for advancing precision prevention strategies in the United States.

### Standardization of microbiome methodology

One of the foremost challenges is the lack of standardized methodologies across microbiome research. Differences in sample collection, sequencing platforms, and bioinformatics pipelines hinder reproducibility and cross-study comparisons [85]. U.S. initiatives, such as the National Microbiome Data Collaborative, are working to harmonize data standards, but broader adoption is needed to ensure robust translation into clinical settings.

### Longitudinal U.S. cohort studies

Most evidence linking the microbiome to obesity and CRC comes from cross-sectional or case–control studies, limiting causal inference. Large-scale longitudinal cohorts integrating microbiome, dietary, and lifestyle data are needed to clarify temporal relationships [86]. Programs like the NIH *All of Us Research Program* and the Nurses’ Health Study provide platforms for such integration, but microbiome data collection remains incomplete. Expanding these efforts will strengthen causal insights and inform public health interventions.

### Microbiome in precision medicine

Microbiome-informed risk stratification and treatment tailoring represent promising avenues for precision medicine. For CRC, microbial signatures may help identify high-risk individuals for earlier colonoscopy screening [87]. In obesity and metabolic disease, microbiome-based stratification may predict which patients will respond best to dietary or probiotic interventions. However, clinical algorithms incorporating microbiome features require prospective validation in diverse U.S. populations.

### Integration with artificial Intelligence and multi-omics

The complexity of microbiome–host interactions necessitates advanced computational tools. Artificial intelligence (AI) and machine learning models can integrate microbiome, metabolome, and host genomic data to predict disease risk [88]. Multi-omics approaches are particularly well-suited for distinguishing functional microbial pathways from taxonomic signals. While early studies show promise, building explainable and clinically deployable AI models remains a critical gap.

### Addressing diversity and health equity

Most microbiome research has been conducted in populations of European ancestry, limiting generalizability to the diverse U.S. population. Differences in microbiome composition across ethnic and socioeconomic groups suggest that one-size-fits-all approaches may exacerbate disparities [89]. Ensuring inclusion of underrepresented groups in microbiome research is essential to achieve equitable public health benefits.

### Ethical, legal, and social implications

Microbiome data collection raises questions about privacy, ownership, and commercialization. Fecal microbiota transplantation and microbiome therapeutics also introduce regulatory and ethical complexities surrounding donor selection, informed consent, and access. Establishing clear frameworks will be necessary to balance innovation with patient protection [90].

## CONCLUSIONS

The gut microbiome has emerged as a central player in shaping host metabolism, immune regulation, and carcinogenesis. Evidence linking dysbiosis to obesity, metabolic syndrome, and colorectal cancer underscores its relevance to two of the most pressing public health challenges in the United States. Microbial composition, metabolite production, and immune modulation collectively create pathways that either promote health or drive disease, offering opportunities for early detection, prevention, and therapeutic intervention.

From stool-based microbial biomarkers to FDA-approved microbiota-based therapeutics, translational advances are moving the field from discovery toward clinical application. At the same time, integration of microbiome science into national initiatives—ranging from dietary guidelines to cancer prevention strategies—has the potential to reshape U.S. public health policy. Ensuring equitable access, methodological standardization, and inclusion of diverse populations will be essential for realizing these benefits at scale.

Looking forward, the convergence of microbiome research with artificial intelligence, multi-omics technologies, and precision medicine offers a pathway toward tailored interventions that can reduce obesity and colorectal cancer burden nationwide. By bridging microbiology, gastroenterology, oncology, and public health, the gut microbiome represents not only a frontier of scientific exploration but also a cornerstone for advancing national health priorities in the United States.
